# A Cost-Effective and Non-Invasive pfeRNA-Based Test Differentiates Benign and Suspicious Pulmonary Nodules from Malignant Ones

**DOI:** 10.3390/ncrna7040080

**Published:** 2021-12-16

**Authors:** Wei Liu, Yuyan Wang, Hongchan Huang, Nadege Fackche, Kristen Rodgers, Beverly Lee, Wasay Nizam, Hamza Khan, Zhihao Lu, Xiangqian Kong, Yanfei Li, Naixin Liang, Xin Zhao, Xin Jin, Haibo Liu, Charles Conover Talbot, Peng Huang, James R. Eshleman, Qi Lai, Yi Zhang, Malcolm V. Brock, Yuping Mei

**Affiliations:** 1 Guangdong Key Laboratory of Liver Disease Research, The Third Affiliated Hospital, Sun Yat-Sen University, 600 Tianhe Road, Tianhe District, Guangzhou 510630, China; Dr.liuwei@outlook.com (W.L.); liyanfei_333@163.com (Y.L.); 2 The Sidney Kimmel Comprehensive Cancer Center, Johns Hopkins School of Medicine, 1650 Orleans Street, Baltimore, MD 21287, USA; wangyuyan1219@aliyun.com (Y.W.); NFackche@huhosp.org (N.F.); kpelosk1@jhmi.edu (K.R.); blee55@alumni.jh.edu (B.L.); wnizam1@jhmi.edu (W.N.); hkhan18@jhmi.edu (H.K.); zhihaolupku@bjmu.edu.cn (Z.L.); kong_xiangqian@gibh.ac.cn (X.K.); laiqi@swmu.edu.cn (Q.L.); 3 Department of Thoracic Medical Oncology, Key Laboratory of Carcinogenesis and Translational Research (Ministry of Education), Peking University Cancer Hospital and Beijing Institute for Cancer Research, 52 Fucheng Road, Haidian District, Beijing 100142, China; 4 State Key Laboratory of Respiratory Disease, Guangzhou Institutes of Biomedicine and Health, Chinese Academy of Sciences, Huangpu District, Guangzhou 510530, China; huang_hongchan@gibh.ac.cn; 5 China-New Zealand Joint Laboratory on Biomedicine and Health, Guangzhou Institutes of Biomedicine and Health, Chinese Academy of Sciences, Huangpu District, Guangzhou 510530, China; 6 Department of Gastrointestinal Oncology, Key Laboratory of Carcinogenesis and Translational Research (Ministry of Education), Peking University Cancer Hospital and Beijing Institute for Cancer Research, 52 Fucheng Road, Haidian District, Beijing 100142, China; 7 Thoracic Surgery Department of Peking Union Medical College Hospital, Dongcheng District, Beijing 100034, China; pumchnelson@163.com; 8 Department of Thoracic Surgery, Lung Cancer Center, Xuanwu Hospital, Capital Medical University, 45 Changchun Street, Xicheng District, Beijing 100053, China; zx89316@163.com (X.Z.); xin.jin@student.kuleuven.be (X.J.); 9 Thoracic Surgery Department of Peking University, The First Hospital, Xicheng District, Beijing 100034, China; lhb118@sina.com; 10 Institute for Basic Biomedical Sciences, Johns Hopkins School of Medicine, 733 North Broadway, Baltimore, MD 21205, USA; cct@jhmi.edu; 11 Department of Oncology, Sol Goldman Pancreatic Cancer Research Center, Johns Hopkins Medical Institutions, Baltimore, MD 21287, USA; phuang12@jhmi.edu; 12 The Pathology Molecular Diagnostics Laboratory, Johns Hopkins School of Medicine, 600 N Wolfe St., Baltimore, MD 21287, USA; jeshlem@jhmi.edu; 13 Department of Thoracic Surgery, Sichuan Cancer Center Hospital & Institute, School of Medicine, University of Electronic Science and Technology of China, Chengdu 611731, China

**Keywords:** pfeRNA, non-invasive biomarker, pulmonary nodules, NSCLC, CLIA, LDT

## Abstract

The ability to differentiate between benign, suspicious, and malignant pulmonary nodules is imperative for definitive intervention in patients with early stage lung cancers. Here, we report that plasma protein functional effector sncRNAs (pfeRNAs) serve as non-invasive biomarkers for determining both the existence and the nature of pulmonary nodules in a three-stage study that included the healthy group, patients with benign pulmonary nodules, patients with suspicious nodules, and patients with malignant nodules. Following the standards required for a clinical laboratory improvement amendments (CLIA)-compliant laboratory-developed test (LDT), we identified a pfeRNA classifier containing 8 pfeRNAs in 108 biospecimens from 60 patients by sncRNA deep sequencing, deduced prediction rules using a separate training cohort of 198 plasma specimens, and then applied the prediction rules to another 230 plasma specimens in an independent validation cohort. The pfeRNA classifier could (1) differentiate patients with or without pulmonary nodules with an average sensitivity and specificity of 96.2% and 97.35% and (2) differentiate malignant versus benign pulmonary nodules with an average sensitivity and specificity of 77.1% and 74.25%. Our biomarkers are cost-effective, non-invasive, sensitive, and specific, and the qPCR-based method provides the possibility for automatic testing of robotic applications.

## 1. Introduction

Lung cancer remains the leading cause of cancer-related deaths in the United States and worldwide [1]. With the growing popularity of CT screening, physicians are increasingly faced with the clinical dilemma of identifying incidental pulmonary nodules in asymptomatic smokers. Although CT can be highly sensitive, it is not specific. Its high false-positive rate leads to additional follow-up procedures, patient anxiety about indeterminate nodules, risk of over-diagnosis, differences in selection criteria, and radiation exposure [2,3]. Unfortunately, even with functional imaging and predictive tools based on state-of-the-art algorithms, confirmation of malignancy by imaging alone remains a diagnostic challenge. While there are integrated prediction models for pulmonary nodules [4,5,6], the considerable overlap in the clinical characteristics makes it difficult for physicians to distinguish patients with benign and malignant pulmonary nodules. Thus, developing and validating a novel strategy rooted in molecular signatures of blood would represent a real step forward in non-invasive biomarkers.

Liquid biopsies are increasingly recognized as important non-invasive strategies for lung cancer [7,8,9]. Both genetic and epigenetic signatures of plasma circulating tumor DNA (ctDNA) have been utilized to develop blood tests for lung cancer. However, the levels of plasma ctDNA are dependent on tumor burden, and fewer than one mutant template molecule per milliliter (mL) of plasma generally exists in patients with early stage lung cancer [10,11,12]. The low sensitivity [13,14,15] or severely compromised specificity [16] of ctDNA-based detection was observed in diagnosing early stage lung cancer and differentiating benign from malignant pulmonary nodules. In addition, the high cost and sophisticated procedures of ctDNA-based tests challenge their feasibility as generalized screening strategies for pulmonary nodules diagnosis, especially in remote and impoverished areas. Therefore, it is desirable to explore other liquid biopsy biomarkers to develop a novel, non-invasive, easy to operate, and cost-effective test for the accurate diagnosis of pulmonary nodules.

Previously, we showed that pfeRNA is a type of unique functional sncRNAs that plays a critical role in the tumorigenesis and differentiation of non-small cell lung cancer (NSCLC) [17,18,19,20]. Without changing the levels of the target protein, pfeRNA directly binds to its target and regulates the functional behaviors of the target protein [17,18,19,20]. Since NSCLC and normal human bronchial epithelial cell lines can be distinctly clustered based on the expression patterns of pfeRNAs [17,18,19], we sought to design a blood-based assay based on pfeRNAs. We hypothesized that plasma pfeRNAs might be used non-invasively to detect the existence and oncologic nature of an indeterminate pulmonary nodule found on CT scans in patients at high risk for lung cancer. Our assay needed to satisfy four criteria. First, the pfeRNAs had to be capable of determining both the existence and the nature of a pulmonary nodule [21,22,23]. Second, the assay had to be composed of a small panel of pfeRNAs appropriately suited for the early detection of pulmonary nodules [21,24]. Third, each pfeRNA in the plasma had to be abundant enough to be detected by QuantStudio real-time PCR with an appropriate reference [25,26,27,28]. Fourth, the assay must be cost-effective for use as a screening tool [24,25,26]. To overcome these challenges, we began by screening differentially expressed pfeRNAs using sncRNA deep sequencing in both plasma and tissue from healthy individuals without any pulmonary disease, from patients with biopsy-proven benign conditions, and from patients with malignant pulmonary nodules. We then initiated a multicenter biomarker study to optimize candidate pfeRNAs, derive prediction rules, and validate the pfeRNAs in independent cohorts. 

## 2. Results

### 2.1. Clinical Features of the Participations

In total, we collected 48 tissue and 488 plasma samples from three groups of participants: healthy controls, patients with benign pulmonary nodules plus suspicious nodules, and patients with malignant pulmonary nodules in Stage-I/II NSCLC. These participants were from four different institutions (Table 1). We used these clinical biospecimens in a retrospective study and randomly distributed them for the discovery stage, training cohort, and validation cohort (Table 1). 

Each cohort included patients with the following clinical descriptors: (i) both genders as well as smokers and non-smokers (Supplementary Table S2), (ii) a healthy control group determined by a physician to be free of any cancer, (iii) a benign group biopsy-proven to be benign, and (iv) a malignant group found free of any other cancer. The histology of the malignant pulmonary nodules included lung adenocarcinoma, squamous cell carcinoma, adenosquamous carcinoma, and large cell carcinoma (Supplementary Figure S1, and Table S3). 

### 2.2. Differentially Expressed pfeRNAs in the Discovery Stage

**Figure 1:**
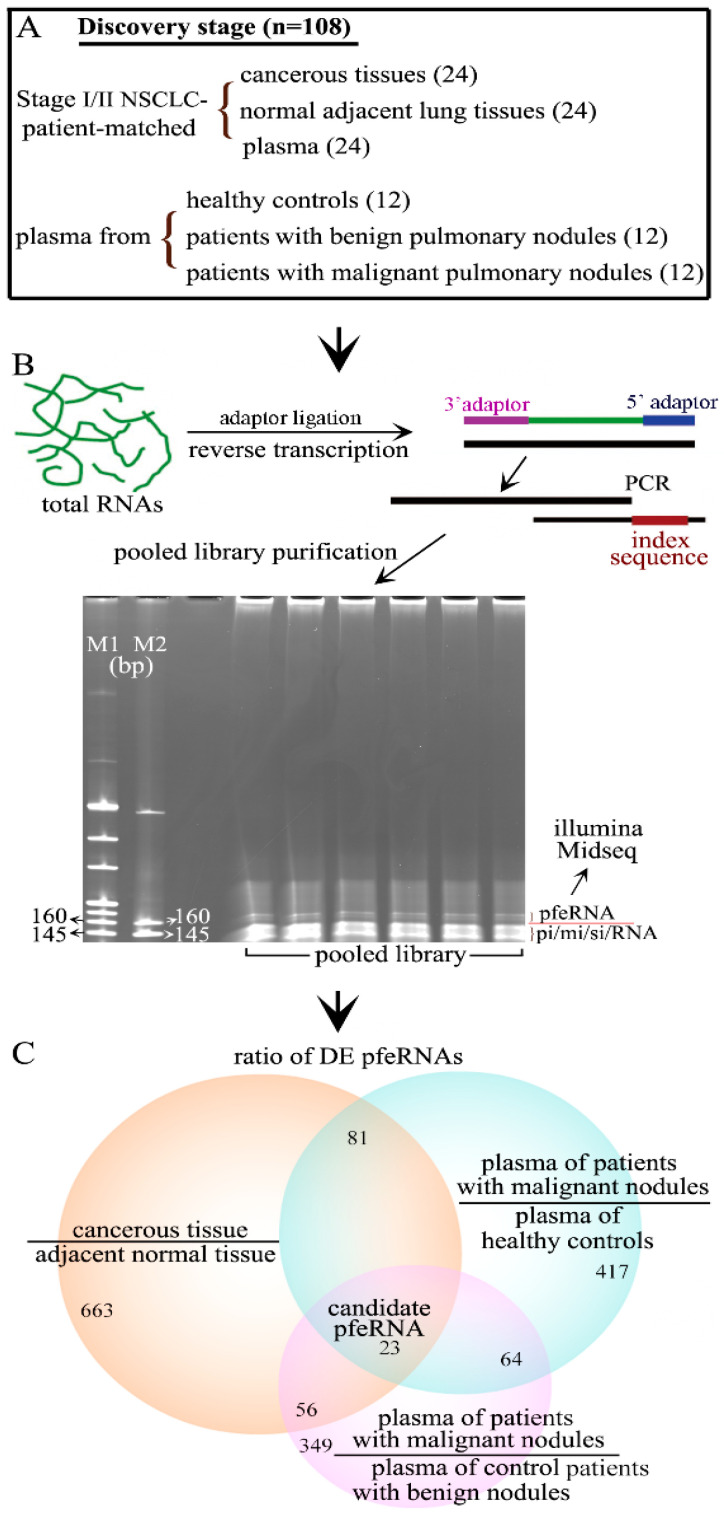
Identifying non-invasive pfeRNA candidates in plasma. (**A**) Biospecimens used in the discovery stage. Upper: 72 patient-matched biospecimens, including cancerous tissues, normal adjacent tissues, and plasma, were from 24 patients with stage I/II NSCLC. Bottom: 36 plasma biospecimens were from 12 healthy controls, 12 patients with benign pulmonary nodules, and 12 patients from malignant pulmonary nodules. (**B**) The preparation process of a pfeRNA library for illumine Midseq. (**C**) Bioinformatics and biostatistics analysis of the differentially expressed (DE) pfeRNAs in different groups.

We used 108 biospecimens from 60 patients in the discovery stage, including 36 patients with biopsy-proven malignant pulmonary nodules in Stage-I/II NSCLC, 12 healthy controls, and 12 patients with pathology-confirmed benign pulmonary nodules (Figure 1A, Table 1). To determine the pfeRNAs that may have oncogenic functions in NSCLC tumorigenesis, we used both cancerous tissue and histologically normal adjacent lung parenchyma from 24 patients with malignant pulmonary nodules in Stage-I/II NSCLC (Figure 1A). We extracted the total RNA, ligated the RNA with 3′ and 5′ end adaptors, performed reverse transcription, used a unique index for each biospecimen, purified the PCR products, and processed the pfeRNA bands for sncRNA deep sequencing (Figure 1B). Using filtered log fold changes in Log_2_FC > 2 and false discovery rate < 0.05 as criteria, we analyzed the differentially expressed pfeRNAs between different groups. First, we found 823 differentially expressed pfeRNAs between malignant tissue (*n* = 24) and the corresponding histologically normal adjacent lung tissue (*n* = 24) (Figure 1C, left). Second, we identified 585 differentially expressed pfeRNAs in the plasma of patients with malignant nodules (*n* = 36) compared to those expressed in plasma from healthy individuals (*n* = 12) (Figure 1C, right). Third, we identified 492 differentially expressed pfeRNAs between the plasma of those with malignant (*n* = 36) versus benign nodules (*n* = 12) (Figure 1C, bottom). Our final analysis revealed that 23 differentially expressed pfeRNAs were common to all clinical patient groups and might serve as non-invasive putative plasma biomarkers for distinguishing both the existence and the nature of pulmonary nodules (Figure 1C).

### 2.3. Non-Invasive pfeRNA Panel

To construct pfeRNA classifiers that could determine both the existence and the nature of pulmonary nodules, we examined the plasma levels of 16 candidates that showed more than a three-fold change between healthy controls, those with benign plus suspicious pulmonary nodules, and patients with malignant pulmonary nodules. We assessed their plasma levels by qPCR in the 108 specimens from 60 patients used in the discovery stage. We found that eight plasma pfeRNAs (pfeRNAa to pfeRNAh, Table 2) exhibited specific amplification in all samples (Supplementary Figures S2 and S3). These pfeRNAs are on chromosome 1, 2, 5, 6, 7, 8, 9, 11, 12, 14, 16, 17, and in the mitochondrial genome, and are 33 to 51 nucleotides in length (Table 2). 

### 2.4. The Performance of the pfeRNA Panel in the Training Cohort

**Figure 2:**
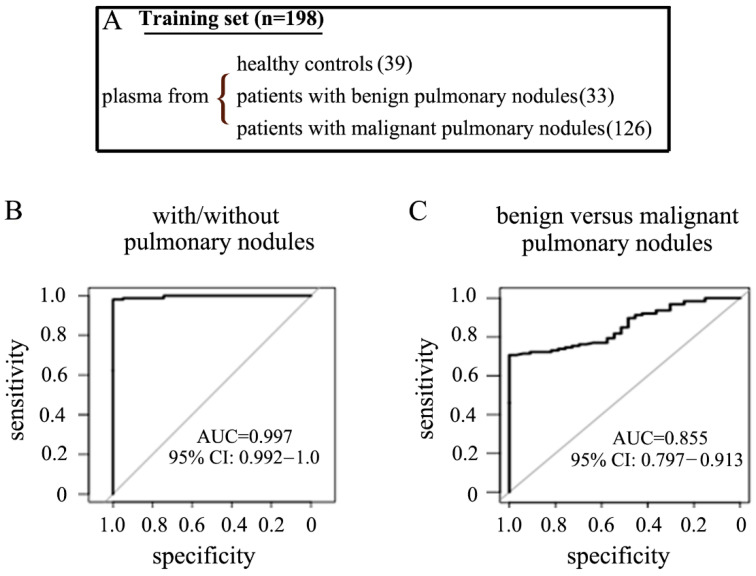
Assay performance in detecting both the existence and the nature of pulmonary nodules in a training cohort. (**A**) Biospecimens used in the training cohort. Receiver operator characteristic (ROC) curves for prediction rules in detecting patients with/without pulmonary nodules (**B**) and patients with benign versus malignant pulmonary nodules (**C**).

Next, we evaluated the expression levels of these 8 pfeRNAs in the plasma of 198 patients from a training cohort, which included 39 healthy controls, 33 patients with biopsy-proven benign pulmonary nodules, and 126 patients with malignant pulmonary nodules (Figure 2A, Table 1). We then derived prediction rules based on the plasma levels of these pfeRNAs (Table 3). Rules derived from these pfeRNAs were able to (1) detect individuals with pulmonary nodules (including benign and malignant nodules) with a sensitivity and specificity of 98.1% and 100%, respectively (Figure 2B left, Table 4), and (2) differentiate patients with biopsy-proven malignant from those with benign pulmonary nodules with a sensitivity and specificity of 76.2% and 69.7%, respectively (Figure 2C right, Table 4). 

### 2.5. The Performance of the pfeRNA Panel in the Validation Cohort

**Figure 3:**
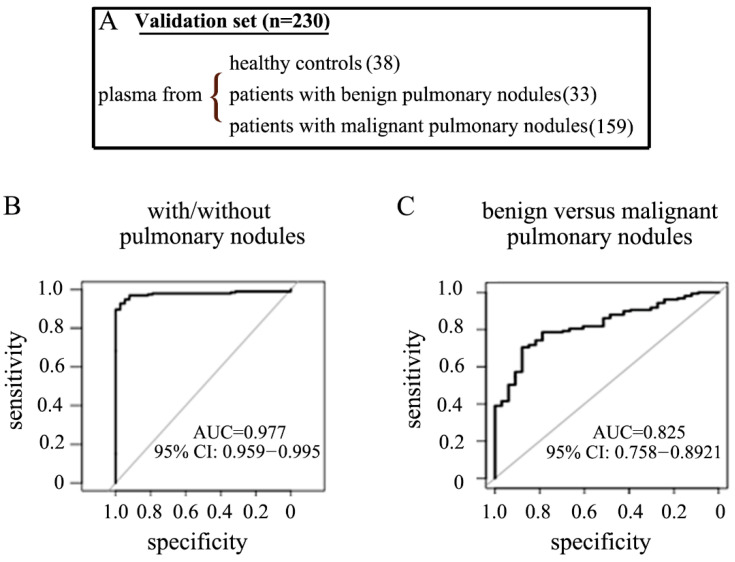
Assay performance in detecting both the existence and the nature of pulmonary nodules in a validation cohort. (**A**) Biospecimens used in the validation cohort. ROC curve for prediction rules in detecting patients with/without pulmonary nodules (**B**) and patients with benign versus malignant pulmonary nodules (**C**).

We then applied these derived rules to an independent validation cohort of 230 patients comprised of 38 healthy controls, 33 patients with benign pulmonary nodules, and 159 patients with malignant nodules (Figure 3A, Table 1). These derived rules allowed us to (1) differentiate patients with and without pulmonary nodules with a sensitivity and specificity of 94.3% and 94.7%, respectively (Figure 3B left, Table 4), and (2) differentiate patients who had malignant versus benign pulmonary nodules with a sensitivity and specificity of 78% and 78.8%, respectively (Figure 3C right, Table 4). 

## 3. Discussion

In current clinical practice, physicians can estimate the probability of malignancy using clinical parameters, nodule size, metabolic, and morphological assessments [29,30,31,32]. The considerable overlap in these clinical characteristics makes it difficult to distinguish patients with benign and malignant pulmonary nodules. In this study, we chose to use plasma from individuals with benign nodules as a rigid control to differentiate from the malignant nodules. The patients with benign nodules in our study were highly suspected of being malignant by their physicians that they underwent pulmonary resection. In the main, these patients were unable to be preoperatively biopsied but ultimately proved to have benign nodules after resection. Compared to studies that only use healthy controls, our utilization of these surgical patients with pathologically proven benign nodules mimicked the real lung cancer screening context, significantly reduced the false positive rate of the test, and provided promising data for future clinical application.

Moreover, we also compared the smoking intensity in pack-years among patients in the different groups since cigarette smoking is a well-known risk factor for lung cancer. In our healthy, benign pulmonary nodule, and malignant pulmonary nodule groups, smokers accounted for 41.55%, 44.44%, and 46.41%, respectively (Supplementary Table S2). There was no significant difference in the percentage of smokers among groups, suggesting that our prediction rules can detect the existence and the nature of pulmonary nodules regardless of smoking history. Nevertheless, in our study, patients with malignant pulmonary nodules had a >61 pack-year history which was significantly higher than that of both the healthy group and benign controls (7.55% versus 1.30% and 1.85%, Supplementary Table S2), consistent with the fact that smoking intensity matters when considering the pathogenesis of lung cancer. 

These non-invasive biomarkers belong to a novel type of functional sncRNA. Consistent with our previous reports [17,18,19,20], they showed different lengths in nucleotides (Table 2). Distinct from other integrated prediction models for pulmonary nodules [33,34,35,36,37], our prediction rules did not integrate other clinical risk factors, including age [38], smoking history [39,40,41], irregular nodule edges [42], emphysema [43,44], fluorodeoxyglucose-PET avidity [45,46], etc. We used FDA-cleared equipment and reagents available for in vitro diagnostic use or for R&D, and methods for evaluating pfeRNAs meeting the requirements for the CLIA-compliant LDT. Our qPCR-based assay only needed 200 μL of plasma, and the estimated cost was less than $15 per sample. This non-invasive and cost-effective advantage may advocate for our assay as an initial screening strategy complementary to LDCT for detecting early stage lung cancer. Limitations of our study are its retrospective nature, the availability of only one independent cohort for validation, and the future need for more cohorts, including nested case-controls, to test our prediction rules. Further clinical validation of our pfeRNA panel in a multicenter prospective trial will be needed.

## 4. Materials and Methods

### 4.1. Participants, Plasma, and Tissues

The study was approved by the Institutional Review Boards for Human Research at each institution and complied with Health Insurance Portability and Accountability Act. Informed consent was obtained from all patients, and peripheral blood was collected after informed consent was obtained and prior to the patients undergoing surgical resection. General demographics, surgical pathology (both benign and malignant pulmonary nodules), and AJCC stage (7th edition) were documented. The healthy controls and patients with benign pulmonary nodules were determined by the physician to be free of any cancer, patients with early stage NSCLC were determined by the physician to be free of any other cancer, and patients with Stage III/IV NSCLC were excluded from the study. All biospecimens were collected from patients without chemotherapy or radiotherapy before operation. Each cohort included both genders, smokers and non-smokers, and the samples of these groups were processed identically.

Plasma preparation: The whole peripheral blood (7.5 mL) was collected in an anticoagulant tube (K_2_EDTA) and was poured very slowly into a 15 mL conical tube with 5 mL of Ficoll-Paque PLUS buffer (Millipore-Sigma, Cat# GE17-1440-02). The layered mixture was centrifuged for 10 min at 3000 rpm at 4 °C, and then the top plasma layer was transferred to 1.5 mL tubes. The actual plasma layer was around 55% of the total blood and was yellowish fluid. If the red cells had been lysed, the plasma appeared pink or red after centrifuge. In such a condition, the sample should not be processed.

### 4.2. CLIA Compliant LDT Assay

Our assay was based on the methods for “Real-time PCR for nucleic acid-based in vitro tests used for medical”. To develop a CLIA-compliant LDT assay for validating pfeRNA levels, we used currently FDA-cleared technologies for use in the clinical laboratory. Based on the methods for the nucleic acid-based existing in vitro assay [24,25,26], we used the equipment and reagents that are already used in in vitro diagnostic testing. The Real-Time PCR machine for evaluating pfeRNA levels was the QuantStudio Dx PCR Instrument at the CLIA-certified Molecular Diagnostics Laboratory at the Johns Hopkins Hospital. Additionally, we used available commercial reagents for in vitro diagnostic use or for R&D: Chloroform (Sigma-Aldrich, Cat# C2432, for R&D), Isopropyl Alcohol (Thermo Scientific Richard-Allan Scientific, for in vitro diagnostic use), and Ethyl Alcohol (Thermo Scientific Richard-Allan Scientific, for in vitro diagnostic use). The personnel evaluating pfeRNA levels were trained and understood all standards, including Good Laboratory Practices and ISO 17025.

### 4.3. Total sncRNAs Extraction

#### 4.3.1. Total RNAs Extracted from Tissues

Tissues (0.3~0.5 g per sample) were cut into small pieces on ice, transferred to tubes with 1 mL of TRIzol Reagent (cat# 15596018), and homogenized using Tissue Tearor (model 985370–395) on ice. To avoid cross-contamination, we washed the head part of the Tearor twice using 75% ethyl alcohol, followed by using nuclease-free water twice (Promega Corporation, cat# 1193) after each sample. Then, the total RNAs were extracted according to the manufacturer’s instructions.

*Total sncRNAs extracted from total RNAs:* For sncRNA separation, the extracted total RNAs were separated using Craig C. Mello Lab’s sncRNA cloning protocols (Gu W. and Conte D.) using the mir-Vana miRNA Isolation Kit (ThermoFisher Scientific, cat# AM1560) with minor modifications. The following reagents were mixed in a 1.5-mL Eppendorf (EP) tube: no more than 80 µL (<1 mg) of total RNA, 400 µL (5× volume of total RNA) of mirVana lysis/binding buffer, 48 µL (1/10 volume of total RNA) lysis/binding buffer. The mixture was incubated at room temperature for 5 min to denature RNA. Then, 1/3 volume (176 µL) of 100% ethyl alcohol was added to it, and the mixture was spun at 2200 g for 4 min at room temperature to remove larger (>200 nt) RNA. The supernatant containing sncRNAs was transferred to a new EP tube, and sncRNAs were precipitated with 700 µL of isopropanol at −80 °C until frozen (~30 min). Finally, the mixture was centrifuged at 20,000× *g* for 20 min. The pellet was washed with 1000 µL of 75% pre-cold ethyl alcohol, and was dissolved in nuclease-free water.

#### 4.3.2. Total sncRNAs Extracted from Plasma

A total of 200 µL of plasma were used for each sample, and 800 µL of TRIzol Reagent (ThermoFisher Scientific, cat# 15596018) were added to the sample. The mixture was vortexed for 15 s at high speed (VWR, Analog Vortex Mixer), kept at room temperature for 10 min, vortexed for another 15 s after 200 µL of chloroform were added to it, and kept at room temperature for another 10 min. The mixture was then centrifuged at 12,000× *g* for 15 min at 4 °C, and the aqueous supernatant was transferred to a new tube. Then, the following reagents were added to the new tube: 700 µL of isopropyl alcohol, 2 µL of glycogen (ThermoFisher Scientific, cat# R0561), and 50 µL of 3M sodium Acetate (PH5.2, Quality Biological, cat# 351035721). The mixture was kept at −80°C until it was frozen (~20 min). Finally, the mixture was centrifuged at 20,000× *g* for 20 min. The pellet was washed with 1000 µL of 75% pre-cold ethyl alcohol, and was dissolved in 20 µL of nuclease-free water.

### 4.4. Prepare pfeRNA Library for Deep Sequencing

The 5′- and 3′-end adaptors containing barcodes were ligated to extracted sncRNAs. Reverse Transcription (RT)-PCR was performed according to the manufacturer’s instructions of the True Small RNA kit (Illumina, cat# 15016911-15016918). Bands of pfeRNAs were purified, and the pooled-library sequencing was performed using an Illumina NextSeq 500 sequencer in the Core Facility of the Institute for Basic Biomedical Sciences at Johns Hopkins University.

### 4.5. RT and QuantStudio Dx PCR

The whole process of the evaluation of pfeRNA expression levels was similar to what we described before [17,18,20], and the process included adaptor ligation, RT, and QuantStudio Dx PCR. The adaptor/5′rapp/5′-CTGTAGGCACCATCAAT-3′/3′ddc/with both 5′ and 3′ modification, meaning only the 3′-end of sncRNA was ligated. Specifically, 5 µL of total sncRNAs and 1 µL (2 µM) of adaptor were ligated using single-strand truncated T4 RNA ligase 2 (New England Biolabs, cat# M0242L) overnight at 16 °C, and the ligation reaction was terminated at 65 °C for 15 min. For RT, the SuperScript II First-Strand Synthesis System (ThermoFisher Scientific, cat# 18064) was used according to the manufacturer’s instructions with gene-specific reverse transcription primer, and the total volume was 20 µL after RT. For QuantStudio PCR, a common reverse primer and primers specific for individual pfeRNA were used, and the amplification quality of each pair of primers was determined by both generating the melting curves and amplification curves. Each sample was tested in triplicate, and the total volume of each reaction was 20 µL. Amplification conditions were denaturation at 95 °C for 15 s (15 min for the first cycle), annealing at 60 °C for 20 s, extension at 72 °C for 20 s, and 40 cycles. All primers and adaptor are listed in Supplementary Table S1.

### 4.6. Quantitation of pfeRNA Levels in Plasma

We strictly controlled the quantity of total sncRNAs for RT and the quantity of templates for QuantStudio Dx PCR. We used 5 uL of total sncRNAs from 20 µL of each plasma sample for RT, and the sncRNAs in 20 µL of cDNA solution after RT. We then used 2 µL of cDNA for each reaction of QuantStudio Dx PCR. We implemented this quantity-control approach for two reasons. First, it generates the similar cycle threshold (CT) values of the loading control among samples and provides a more reasonable comparison analysis, and second, its results are repeatable in analysis.

Additionally, we used two different spike-in non-human sncRNA as references. One was the well-known cel-miR-67-3p (Abm, Cat# MPH00008 and MCH00003), and the other was the non-human reference with a similar size as pfeRNAs as another spike-in positive control for evaluating RT efficiency. Spike-in positive controls and no template negative controls were included, and pfeRNAs with an undetectable number of qPCR cycles were assumed to have their expression at 40 cycles. pfeRNA levels were normalized against the level of a reference by 2^ΔCT^, where ΔCT = CT_reference_ − CT_target_ [47,48].

While the spike-in non-human sncRNAs as loading controls have been accepted for qualifying the relative expression levels of sncRNAs in the peripheral blood system [27,28], the spike-in reference only served as a loading control but not a real internal reference, because it was not an endogenous control from the human plasma but was artificially added. Thus, the spike-in reference could not serve as a real endogenous reference. However, our prediction rules were derived from the relative expression levels of eight pfeRNAs, the level of one pfeRNA in the rules was always related to the other one of eight pfeRNAs, and the effects of the spike-in reference were canceled out when the relative levels of two pfeRNAs were calculated, For example, the expression levels of pfeRNAa = 2^CTreference − CTpfeRNAa^, and pfeRNAb = 2^CTreference − CTpfeRNAb^, then the relative expression levels of pfeRNAa to that of pfeRNAb was pfeRNAa/pfeRNAb = 2^(CTreference − CTpfeRNAa)^/2^(CTreference − CTpfeRNAb)^ = 2^(CTreference − CTpfeRNAa) − (CTreference − CTpfeRNAb)^ = 2 ^(CTpfeRNAb − CTpfeRNAa)^.

Thus, the results remained the same with or without spike-in references, providing more reasonable and convincible data.

### 4.7. Bioinformatics Analysis

#### 4.7.1. Sequences from sncRNA Deep Sequencing

The sequencing reads were utilized to identify a set of unique sequences for each sample using an auxiliary script from the miRDeep2 software package, and the unique set of sequences were then aligned to the human NCBI reference (build GRCh38) using the QIAGEN CLC Genomics Workbench 10.1.1 software package to determine the genomic locus and the relative number of transcripts for each RNA sequence. The Partek Genomics Suite v7.0 and TIBCO Spotfire DecisionSite v9 platforms were utilized for the analysis of differentially expressed known and novel sncRNAs. The selected differentially expressed pfeRNAs were utilized for downstream visualization and analysis as we did before [17,18]. Differentially expressed pfeRNAs among groups were generated based on the sncRNA deep sequencing. Significance analysis of sequences was applied to identify candidate pfeRNAs with differential levels among groups to ensure that the difference was deemed to be clinically meaningful, and the candidate pfeRNAs to be non-invasive biomarkers in plasma.

#### 4.7.2. Statistical Analysis for the Prediction Rules

In the training set, a classification tree was used to identify pfeRNA biomarkers that best differentiate healthy controls from patients with benign or malignant pulmonary nodules. A logistic regression model with identified pfeRNA biomarkers as covariates was fitted using the training sample. The model was then applied to the validation sample to predict the probability of whether an individual has a pulmonary nodule. The prediction accuracy was evaluated using the area under the receiver operating characteristic (ROC) curve (AUC) and its 95% confidence interval using pROC package. All computations were implemented in Rstudio.

## 5. Conclusions

Plasma pfeRNAs could be non-invasive biomarkers for the early detection of patients with NSCLC. These pfeRNAs can identify both the existence and nature of pulmonary nodules with a high degree of sensitivity and specificity and may represent a novel method to reduce misdiagnosis of lung cancer.

## 6. Patents

This study was performed under the U.S. Patent Number 10,899,812 “Short Non-Coding Protein Regulatory RNAs and Methods of Use”.

## 7. Translational Relevance

We developed our test using the CLIA-compliant LDT methodology, which highlights the translational potential of our non-invasive assay. The test could be used to routinely screen patients for benign pulmonary nodules with suspicious and malignancy, and ultimately, provide patients with a chance of curative resection while avoiding overdiagnosis or overtreatment. Except for its cost-effectiveness and non-invasiveness, our test has two more features. First, the test differentiates patients with and without lung nodules, providing an important clinical assay needed in remote impoverished areas of the world with a high prevalence of smokers but no access to CT scanning. Second, the test differentiates patients who have malignant versus benign lung nodules, providing a novel manner for economically advanced countries enrolling smokers in CT lung cancer screening programs.

## Figures and Tables

**Table 1:** Distribution of clinical biospecimens in different cohorts.

Cohort	Institution	Plasma from Healthy Persons	Plasma from Patients with Benign Pulmonary Nodules	Plasma from Patients with Malignant Pulmonary Nodules in Stage-I/II NSCLC	Normal Tissues from Patients with Malignant Pulmonary Nodules in Stage-I/II NSCLC	Cancerous Tissues from Patients with Malignant Pulmonary Nodules in Stage-I/II NSCLC
Discovery cohort	Cancer Center of JHU		12	12		
	Xuanwu Hospital	12		24	24	24
Training cohort	Cancer Center of JHU	12	17	56		
	Xuanwu Hospital	17	11	40		
	The Third Affiliated Hospital of SYU	10		16		
	Peking Union Medical College Hospital		5	14		
Validation cohort	Cancer Center of JHU		23	53		
	Xuanwu Hospital	30	4	90		
	The Third Affiliated Hospital of SYU	8				
	Peking Union Medical College Hospital		6	16		

**Table 2:** Sequences of pfeRNAs and their distribution in chromosomes (Chr).

pfeRNA	Sequence (5′–3′)	Genomic Location
pfeRNAa	TAAAGTTGGTATACAACCCCCCACTGCTAAATTTGACTGGCTT	Genomic chr 1, 7, 8, 9, 12 and 17
pfeRNAb	ATTGGTCGTGGTTGTAGTCCGTGCGAGAATACCA	Genomic chr 13 and X
pfeRNAc	TAGCTTATCAGACTGATGTTGACTGTTGAATCTCATGGCAACACCAGTT	Genomic chr 5
pfeRNAd	GGCTGGTCCGATGGAAGTGGGTTATCAGAACTAATTAACTT	Genomic chr 2 (reverse strand), 6 and 7
pfeRNAe	TCGGATCCGTCTGAGCTTGGCTGCCCGGCTAGCTCAGTCGGTAGAGCATGA	Genomic chr 1, 5, 6, 14, and 16
pfeRNAf	AAGCACCCAACTTACACTTAGGAGATTTCAACTTAACTTGACCGCTCTGACCA	Genomic chr 7 and Mitochondria
pfeRNAg	GGCTGGTCCGATGGTAGTGGGTTATCAGAACTTATTAACT	Genomic chr 6 and 7
pfeRNAh	TAGGATGGGTGTGATAGGTGGCACGGAGAATTACCAAA	Genomic chr 1 and Mitochondria

**Table 3:** Prediction rules.

To Detect a Candidate with or without Pulmonary Nodule(s)
Rule 1 = (−0.65)*A.H + 0.15*B.F − 0.1*C.H + 0.24*D.F + 0.37*E.F − 0.06*F.G − 0.42
If Rule 1 > 0, classify it to be pulmonary nodules (benign + malignant nodules)
If Rule 1 ≥ 0, classify it to be healthy
To detect benign versus malignant pulmonary nodules
Condition 1 = −0.0900*A.F−0.0607*C.H + 0.0545*F.G − 0.0050*H.F + 1.3508*(F.G ≥ −1.8857)
Condition 2 = −0.0136*A.F−0.0223*C.H + 0.9837*( R1 ≥ 0.1794) + 0.6496*(condition 1)
Rule2 = −0.0569*A.F − 0.0141*B.E + (−0.0434)*B.H + (−0.0847)*C.D − 0.0420*C.H + (−0.0282)*D.E − 0.0621*H.F + 1.1040*( condition 1) ≥ 0.1794) + 0.4962*(condition 2) +1
If Rule 2 > 0, classify it to be malignant pulmonary nodules
If Rule 2 ≤ 0, classify it to be benign pulmonary nodules

**Table 4:** Performance of pfeRNAs for detecting healthy controls, patient controls with benign, and patients with malignant pulmonary nodules.

	Sensitivity (%)	Specificity (%)
Training cohort		
With versus without pulmonary nodules	98.1	100
Malignant versus benign pulmonary nodules	76.2	69.7
Validation cohort		
With versus without pulmonary nodules	94.3	94.7
Malignant versus benign pulmonary nodules	78	78.8

## Data Availability

All data generated or analyzed during this study are included in this published article and its supplementary information files.

## Supplementary Materials

The following are available online at https://www.mdpi.com/article/10.3390/ncrna7040080/s1. Figure S1: Representative consecutive pictures on early-stage NSCLC of patients screened by CT (first line) and LDCT (second line), Figure S2: The representative Amplification Plot and Melt Curve Plot of pfeRNA a-d in different plasma, Figure S3: The representative Amplification Plot and Melt Curve Plot of pfeRNA e-h in different plasma, Table S1: Sequences of primers and adaptors, Table S2: Smoking intensity in pack-year among different groups, Table S3: Percentage of different subtypes of NSCLC.
