# Supplementary material for: A Cost-Effective and Non-Invasive pfeRNA-Based Test Differentiates Benign and Suspicious Pulmonary Nodules from Malignant Ones
Source: Noncoding RNA. 2021 Dec 16;7(4):80. doi: 10.3390/ncrna7040080 (PMC8709422; doi:10.3390/ncrna7040080)
Supplement: Supplementary file 1 [file ncrna-07-00080-s001.zip › ncrna-1491920-supplementary.pdf]

Supplementary information: The supplementary data includes Figure S1 to Figure S3, Table S1 to Table S3.

Figure S1: Representative consecutive pictures on early-stage NSCLC of patients screened by CT (first line) and LDCT (second line).

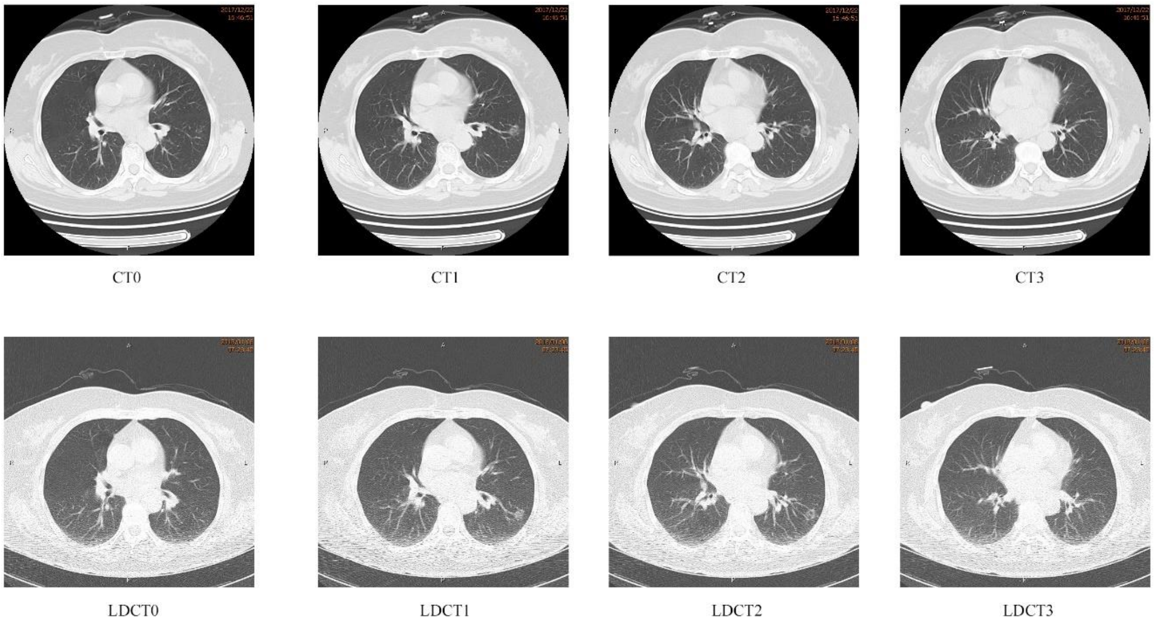

Figure S2: The representative Amplification Plot and Melt Curve Plot of pfeRNA a-d in different plasma.

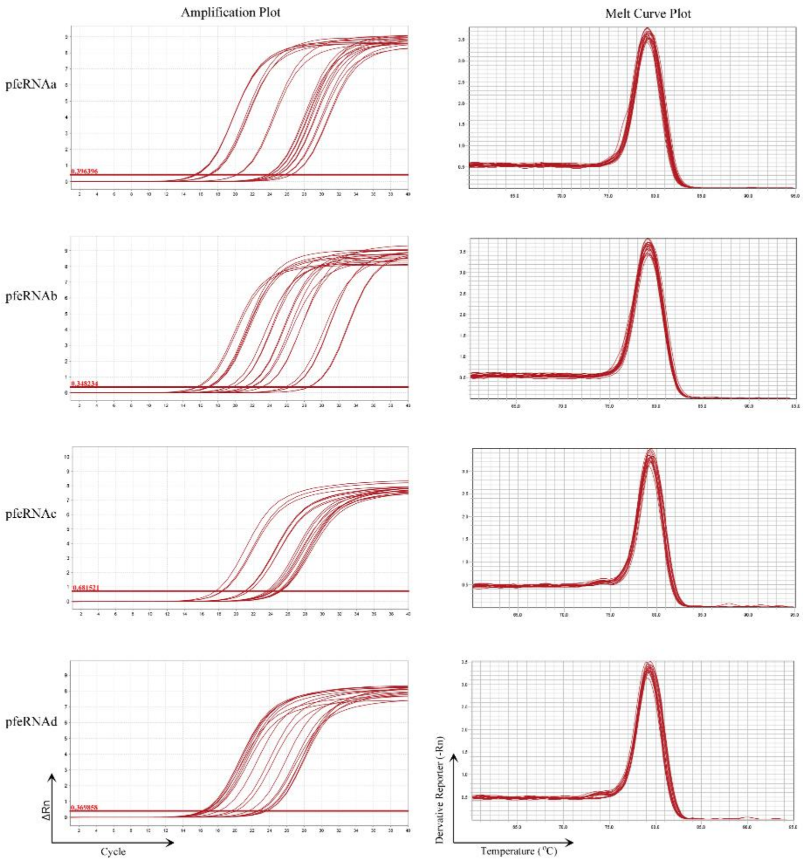

Figure S3: The representative Amplification Plot and Melt Curve Plot of pfeRNA e-h in different plasma.

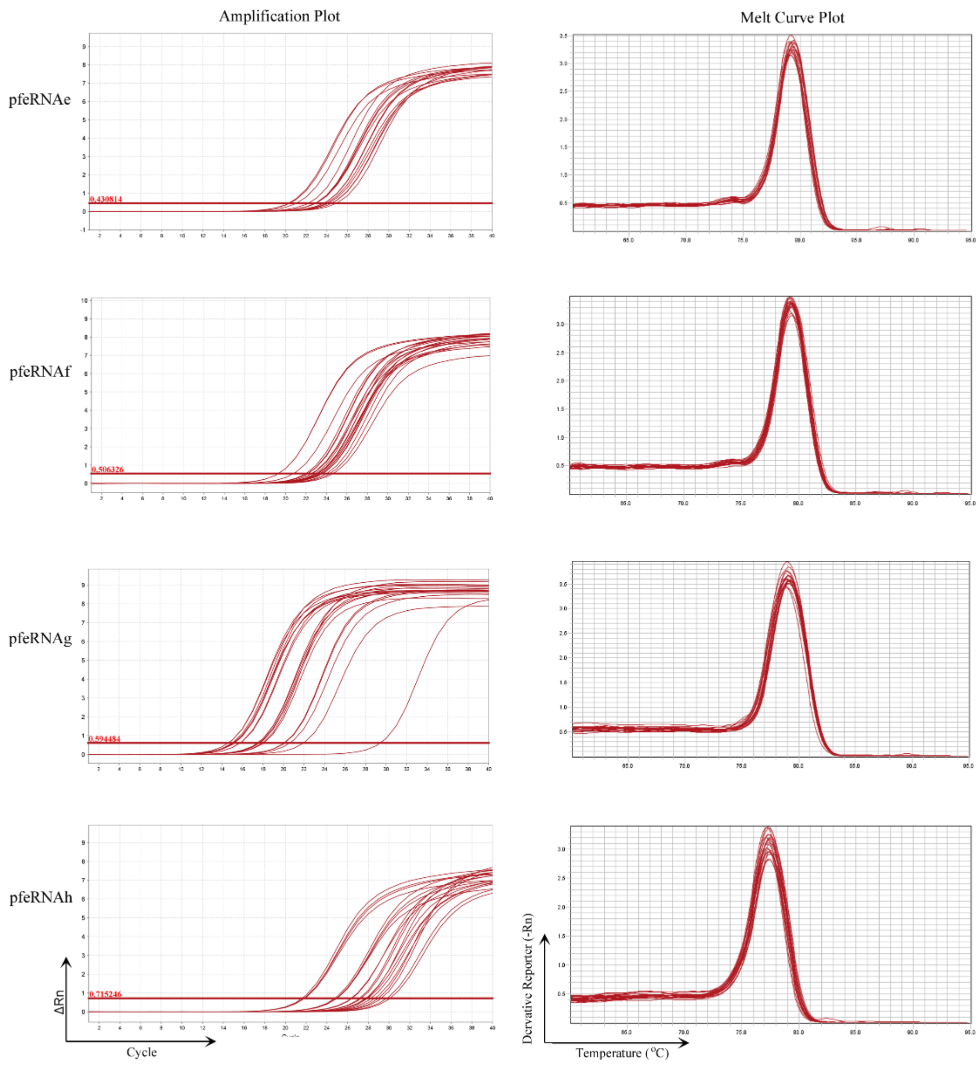

Table S1: Sequences of primers and adaptors

Name	sequences (5’ to 3’)	application
3' adaptor	rapp/5'-CTGTAGGCACCATCAAT-3'/3'ddc	ligation
gene-specific primer	CAAGCAGAAGACGGCATACGATTGATGGTGCCTACAG	Reverse Transcription
common reverse primer	CAAGCAGAAGACGGCATACGA	RT-PCR
pfeRNAaF	CACTGCTAAATTTGACTGGCTT	qPCR
pfeRNAbF	CGTGGTTGTAGTCCGTGCGA	qPCR
pfeRNAcF	TAGCTTATCAGACTGATGTTGA	qPCR
pfeRNAdF	ATGGAAGTGGGTTATCAGAAC	qPCR
pfeRNAeF	CGGATCCGTCTGAGCTTGGC	qPCR
pfeRNAfF	CAACTTAACTTGACCGCTCTGACCA	qPCR
pfeRNAgF	GCTGGTCCGATGGTAGTGGG	qPCR
pfeRNAhF	GGGTGTGATAGGTGGCACGG	qPCR

Table S2: Smoking intensity in pack-year among different groups

Pack years	1~10	11~20	21~30	31~40	41~50	51~60	>61	percentage of smokers
Healthy controls	5.20%	9.09%	6.49%	7.79%	6.49%	5.19%	1.30%	41.55%
Controls with benign nodules	5.56%	7.40%	9.26%	11.11%	5.56%	3.70%	1.85%	44.44%
Patients with malignant nodules	6%	7.17%	8.30%	8.68%	5.28%	3.40%	7.55%	46.41%

Table S3: Percentage of different subtypes of NSCLC

	Adenocarcinoma	Squamous	Adenosquamous	Large Cell
Discovery cohort	18	16	2	
Training cohort	94	30	2	
Validation cohort	113	41	3	2
Total	225	87	7	2
Percentage	70.09%	27.10%	2.18%	0.62%
